# Encapsulation of Ibuprofen by Pickering-Stabilized Antibubbles

**DOI:** 10.1021/acsomega.4c10244

**Published:** 2025-01-22

**Authors:** Charalampos Tsekeridis, Paloma Manuelle Marques da Silva, Guilherme B. Strapasson, Albert T. Poortinga, Heloisa Nunes Bordallo

**Affiliations:** † Niels Bohr Institute, University of Copenhagen, Copenhagen DK-2100, Denmark; ‡ Department of Chemistry and Nanoscience Center, University of Copenhagen, Copenhagen DK-2100, Denmark; § Department of Mechanical Engineering, Polymer Technology, Eindhoven University of Technology, Eindhoven, AZ 5612, Netherlands

## Abstract

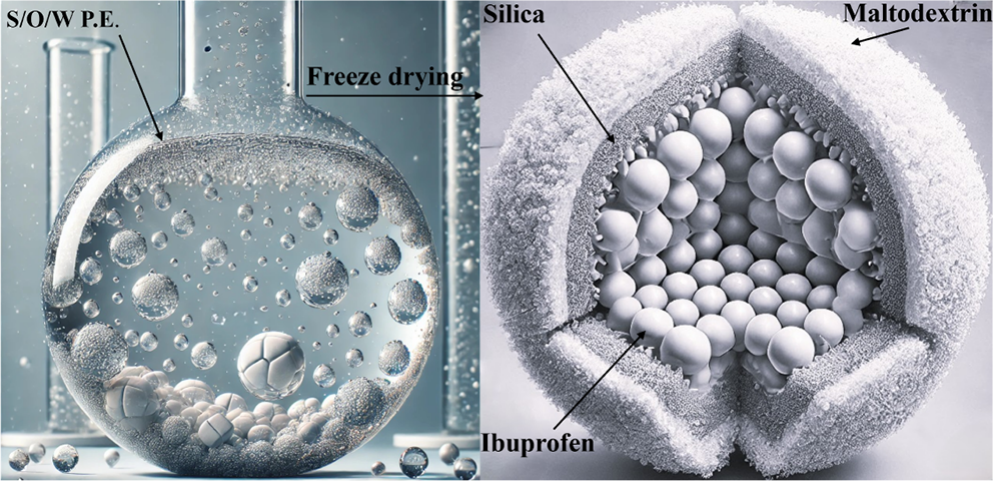

Ibuprofen, one of the most widely used nonsteroidal anti-inflammatory drugs, is a poor-tasting and poorly soluble drug. As an alternative approach to overcome these issues, ibuprofen was encapsulated in Pickering antibubbles using two different oils, cyclomethicone and cyclooctane, as processing aids. The amount of the loaded active agent was determined by thermogravimetry (TG), while the analysis of the evolved gases, performed by online coupling of the heating device to an infrared and a mass spectrometer (EGA-FTIR-MS), allowed for describing the drug decomposition mechanism. Although the dissolution profile and zeta potential values were found to be independent of the preparation method, differential scanning calorimetry (DSC), X-ray powder diffraction (XRPD), and Raman microscopy confirmed the occurrence of a slight amorphization of the drug inside the antibubbles. The reported results suggest that this relatively simple encapsulation technique might be an alternative for ibuprofen taste masking and targeted delivery.

## Introduction

Even though ibuprofen, (*R*,*S*)-2-(4-(2-methylpropyl)phenyl)propanoic acid, is one of the most widely used nonsteroidal anti-inflammatory drugs (NSAIDs),^1,2,4^ it is still the focus of widespread research into the development of smarter formulations for targeted or localized delivery, as well as for taste masking.^3,4^ To this end, microencapsulation, a technology wherein particles are enclosed within a core material, surrounded by a protective shell, is widely used.^5−8^ Indeed, several encapsulation methods, including emulsification, extrusion, spray drying, freeze-drying, and coacervation, have been reported, providing microparticles in the range of a few to a hundred microns.^6,9^ However, obtaining homogeneous mixtures during such formulation processes can be challenging;^10,11^ therefore, as an alternative strategy, we propose the use of antibubbles, defined as bubbles containing one or more cores that can either be solid or liquid.^12^ Although it has been shown that antibubbles significantly improve in vitro drug delivery,^13^ until recently, a real challenge with this method was their short lifespan of about 1 min when dispersed in water. This issue has been, however, solved through the application of the so-called Pickering stabilization, which increased the lifespan to at least 7 days under the same conditions.^14,15^ This process involves generating a water-in-oil-in-water (W/O/W) double emulsion, stabilized by hydrophobized silica particles, where the two water phases contain a solute that solidifies upon drying and the oil phase is volatile. Water and oil are removed from the W/O/W emulsion through freeze-drying, and reconstituting the freeze-dried material in water leads to the formation of the desired antibubbles.^16−18^

In this work, we used a slightly modified method in the sense that ibuprofen crystals were not first dissolved in water, due to its lower solubility of around 11 μg/mL,^19^ but were directly dispersed in the oil phase, using either cyclooctane or cyclomethicone D4, to create a so-called solid-in-oil-in-water emulsion (S/O/W). To study the bulk properties of the encapsulated ibuprofen in the two different oils in the dry state, we employed thermal analysis techniques, X-ray powder diffraction (XRPD), and Raman confocal microscopy. Additionally, the zeta potential and the dissolution profile of the formulations were compared to those of the pure drug.

Thermal analysis of encapsulated pharmaceuticals^5,20,21^ is an excellent analytical methodology to evaluate the properties of composite materials, giving valuable insights into their stability, reactivity, and diverse thermodynamic characteristics. Particularly, data collected through evolved gas analysis (EGA) by integrating mass spectrometry (MS) and Fourier-transform infrared spectroscopy (FTIR)^22^ with thermogravimetric (TG) facilitates the identification of gaseous species produced during thermal degradation, resulting in a comprehensive analysis of the drug release process and decomposition mechanism.^23−25^ On the other hand, by combining differential scanning calorimetry (DSC) with XRPD, it is possible to assess the degree of crystallinity of the studied samples. Furthermore, 2D maps acquired using Raman microscopy, a technique utilizing scattered light to probe the molecular composition within an irradiated volume, allow for a clear label-free spatial representation of drug distribution within the matrix.^26^ Here, this approach gave valuable insight into the intricate interactions between ibuprofen, the sugar matrix covering the antibubbles and the micron-sized antibubbles.

## Methods

### Materials

**Figure 1:**
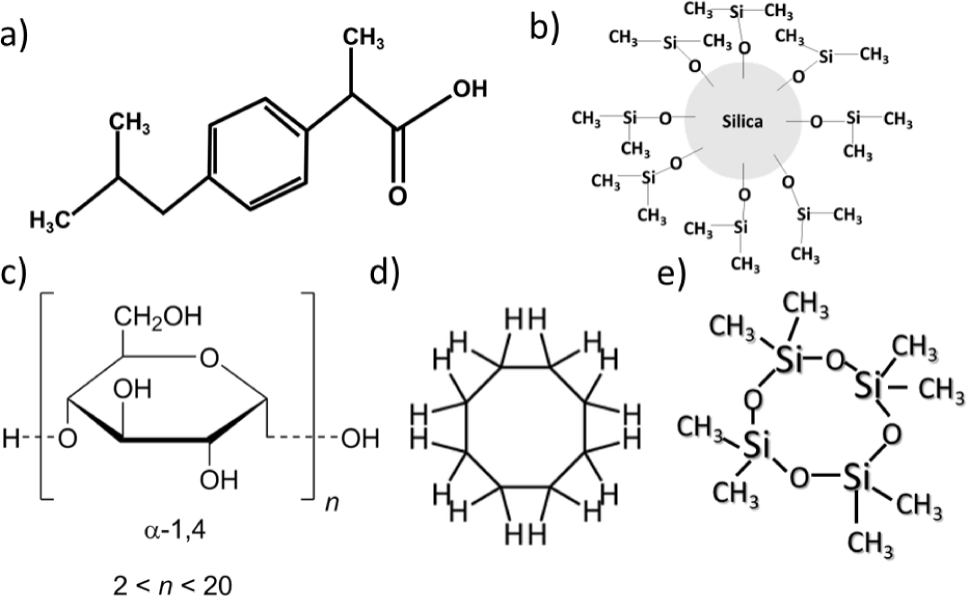
Chemical Structure of the active pharmaceutical ingredient, ibuprofen, (a) and of the excipients used for preparing the microencapsulation system: Aerosil R972 (b), maltodextrin-glucidex 2 (c), cyclooctane, CO (d), and cyclomethicone, CM (e).

Ibuprofen, donated by BASF (Germany), Aerosil R972, a hydrophobized fumed silica particle from Evonik (Germany), maltodextrin-glucidex 2, cyclooctane (CO), and cyclomethicone D4 (CM) from Sigma-Aldrich were used without further purification (Figure 1).

### Preparation of the Antibubbles

Since ibuprofen has poor water solubility,^19^ we made a solid-in-oil-in-water (S/O/W) emulsion by dispersing ibuprofen crystals either in CM or in CO. For CM, the solid-in-oil (S/O) dispersion was obtained using ibuprofen at a concentration of 10 wt % in oil containing 5 wt % hydrophobized silica R972 particles. This sample is hereafter named Ibuprofen_on_ABS_CM. For CO, 5 wt % of ibuprofen and 5 wt % hydrophobized silica R972, hereafter Ibuprofen_on_ABS_CO, were used. For this sample, ibuprofen was visually found to completely dissolve in the oil, thus creating an S/O dispersion. Concurrently, an aqueous phase was produced by dissolving 20 wt % maltodextrin and dispersing 0.5 wt % hydrophobized silica R972 in 20 g of water. Next, the ibuprofen dispersion and the solution were emulsified at a concentration of 20 wt % in the aqueous phase using an Ultraturrax T18 homogenizer (IKA-Werke, Germany) at a speed of 8000 rpm. The resulting particle-stabilized S/O/W emulsions, Ibuprofen_on_ABS_CM and Ibuprofen_on_ABS_CO, were frozen at −60 °C for 24 h and placed in a vacuum freeze-drier (Martin Christ, Alpha 1–2 LDplus, Germany) at a chamber with pressure down to 0.2 mbar. After 24 h, a powder was obtained from the freeze-drying process.^27^

**Figure 2:**
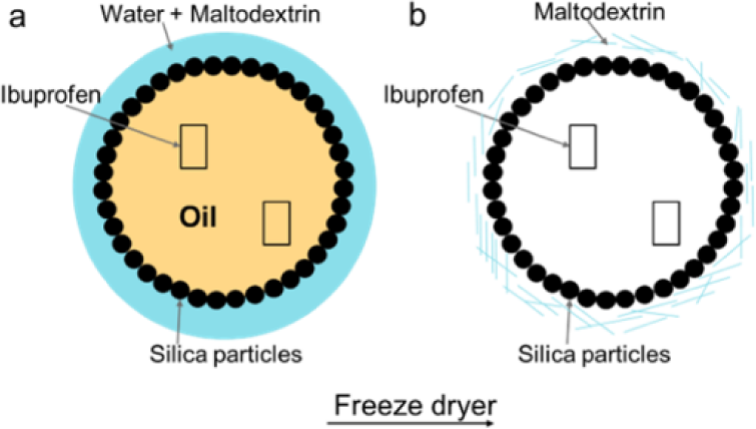
Schematic representation of the two-step process used in the production of ibuprofen encapsulated in the silica antibubbles. (a) A solid-in-oil-in-water (S/O/W) emulsion containing oil droplets that include ibuprofen in crystalline form in the case of cyclomethicone. When cyclooctane is used, ibuprofen completely dissolves in the oil, and hence ibuprofen particles will form during the removal of the oil in the process of freeze-drying. (b) Antibubbles freeze-dried powder obtained after removing the oil and the water. R972 particles were also present in the oil phase and thus in the gas phase, forming a kind of network in which the crystals are entrapped.

This two-step process is shown in Figure 2, where the formation of the (S/O/W) emulsion with the drug inside is depicted in Figure 2A, and the freeze-dried system is depicted in Figure 2B. This pictorial concept is later demonstrated using Raman imaging. Finally, this dry material was reconstituted in an aqueous solution to create a suspension containing entrapped ibuprofen. Based on the composition of the samples, ibuprofen content is expected to be 10.3% in Ibuprofen_on_ABS_CM and 5.4% in Ibuprofen_on_ABS_CO.

### Evolved Gas Analysis (EGA)

Thermogravimetric (TG) analysis coupled with mass spectrometry (MS) and infrared spectroscopy (FTIR) was conducted using a Netzsch TG 209 F1 Libra (Netzsch, Germany) apparatus, simultaneously coupled with an Aëolos QMS403C mass spectrometer and a Bruker spectrometer on dried Pure_Maltodextrin, Pure_Ibuprofen, antibubbles without encapsulated ibuprofen (denoted as ABS_R972), Ibuprofen_on_ABS_CM, and Ibuprofen_on_ABS_CO using an automatic sample changer. FTIR and MS data acquisition were controlled by the OPUS (Bruker, Germany) and Aëolos (Netzsch, Germany) software. Infrared data were acquired every 3 min in the range of 400–4500 cm^–1^, with a resolution of 4 cm^–1^. The quadrupole mass spectrometer (QMS) detected ion currents from the gaseous products over a range of 1–50 *m*/*z*.^25^ To prevent condensation of desorbates within the transfer lines between the TG instrument, the mass spectrometer, and the FTIR spectrometer, these lines were maintained at a temperature of 280 °C.

TG characteristics were derived by expressing weight loss on heating as a percentage relative to the initial mass using Proteus software (Netzsch, Germany). The first derivative of TG (DTG) was computed to determine the temperature corresponding to the most pronounced mass loss, while the mass spectra were normalized to the sample mass. The compounds of the evolved gases were identified by their reference spectra, available in the spectral libraries of NIST.^28^ To account for sample variability, data were collected in triplicate using sample masses between 2 and 4 mg under 20 mL/min of nitrogen as the protective gas and an additional 20 mL/min of Ν_2_ as the purge gas. Samples were placed in Al_2_O_3_ crucibles, the initial temperature was set to 28 °C, and a controlled rate of 5 K/min was used to heat the samples. The maximum heating temperature was selected based on the degradation temperature of the pure compounds,^29−33^ thus avoiding contamination of the FTIR and MS apparatus. In more detail, Pure_Maltodextrin measurements were carried out by heating up to 320 °C, ABS_R972, Ibuprofen_on_ABS_CM, and Ibuprofen_on_ABS_CO up to 300 °C, and Pure_Ibuprofen up to 250 °C. To account for any balance drift in the instrument due to increasing heat, each TG measurement was initiated with a buoyancy correction run, conducted on an empty crucible. This correction mirrored the gas flow and thermal ramp conditions.

### Differential Scanning Calorimetry (DSC)

Data were collected for Pure_Ibuprofen, Ibuprofen_on_ABS_CM, and Ibuprofen_on_ABS_CO using a DSC instrument, Netzsch 214 Polyma, and analyzed using the Proteus software (Netzsch, Germany). Samples with similar masses were sealed in aluminum crucibles with a punched lid in a closed system and measured between approximately −100 and 100 °C under a liquid N_2_ atmosphere purged at 40 mL/min, and the protective gas was 60 mL/min N_2_ with heating and cooling rates of 5 K/min. Before the experiment, an empty crucible and a reference crucible were measured as a correction to adjust the baseline.

### X-Ray Powder Diffraction (XRPD)

Powder diffraction data for pure silica (R972), ABS_R972, Pure_Ibuprofen, maltodextrin, Ibuprofen_on_ABS_CM, and Ibuprofen_on_ABS_CO were collected on a D8 Advance (Bruker, Germany) with Bragg–Brentano configuration using a conventional X-ray source (CuKα line, λ = 1.5406 Å). All measurements were carried out for 1 h at room temperature in a 2θ range from 5° to 70° with increments of 0.01°.

### Raman Spectroscopy and Imaging

Data on Pure_Ibuprofen, Pure_Maltodextrin, Ibuprofen_on_ABS_CM, and Ibuprofen_on_ABS_CO were collected using the inVia confocal Raman microscope and Wire 5.4 software (Renishaw, UK). Spectra were obtained using a 785 nm laser excitation, with laser power varying from 5 to 50 mW and a diffraction grating of 600 lines/mm. The charge-coupled device (CCD) detector (Renishaw Centrus 2N9Y88) allowed covering a spectral range of 100 to 3200 cm^–1^ for ibuprofen and 198 to 2442 cm^–1^ (centered on 1450 cm^–1^) for Ibuprofen_on_ABS_CM and CO with a spectral resolution of ±1 cm^–1^. A Leica DM2700 upright optical microscope with a 50× lens (numerical aperture = 0.5) gave a spatial resolution of 1.92 μm. Acquisition settings were optimized with an integration time of 2 s for Ibuprofen_on_ABS_CM and CO or 10 s for Pure_Ibuprofen). Raman imaging of Ibuprofen_on_ABS_CM covered 121 × 141 μm with 17061 data points.^34^ The full measurement lasted about 10 h.

Data processing was executed using the Wire version 5.4 software and involved cosmic ray removal through a nearest neighbor algorithm. When comparing the full spectra of the four samples, we noticed that the spectra of Pure_Ibuprofen and Pure_Maltodextrin displayed an almost flat background, while the samples with the encapsulated drug showed an increase in the background. Thus, the Raman spectrum of Aerosil R972 was collected, and the observed signal was attributed to fluorescence. With this input, a baseline using the intelligent polynomial algorithm of order 11 for Ibuprofen_on_ABS_CM and Ibuprofen_on_ABS_CO and of order 4 for Pure_Ibuprofen and Pure_Maltodextrin was subtracted. To obtain the map, full principal component analysis (PCA) was performed.

For complementarity, Raman spectra for all samples were also measured using the 785 nm laser excitation with a diffraction grating of 1200 lines/mm and are shown in Figures S1–S3. A spectrum of Pure_Ibuprofen without cosmic ray removal or baseline subtraction is shown in Figure S4.

### Zeta Potential

Using a Zetasizer Pro (Malvern Instruments, UK), measurements were conducted to compare Pure_Ibuprofen, pure silica R972, ABS_R972, Ibuprofen_on_ABS_CM, and Ibuprofen_on_ABS_CO. The samples were prepared by diluting the suspension in distilled water in a ratio of 1:10 (V:V), placed in polystyrene cuvettes, and inserted into the analysis chamber at a temperature of 25 °C. Due to the nature of the sample, we could visually observe that the system is polydisperse and showed large particles. Data analysis was automatically performed using Zetasizer Software.lnk 7.13 (Malvern Instruments, UK).

### In-Vitro Dissolution Studies

Following the recommendations of the International Pharmaceutical Federation,^35^ values for Pure_Ibuprofen were compared to ibuprofen encapsulated in the antibubbles. For all samples, the amount of ibuprofen was fixed at 50 mg. The USP apparatus 2 was used with 250 mL of phosphate buffer (pH = 6.8). The dissolution medium temperature was constantly maintained at 37 °C, and the stirring speed was set to 50 rpm. At different time intervals, 2 mL samples were withdrawn and immediately filtered through a 0.45 μm filter. The absorbance of the dissolved samples was measured via a UV spectrophotometer (Epoch 2 microplate spectrophotometer, BioTek, USA) at 264 nm for each sample. The percentage of drug dissolved was calculated by the classic method of using an extrapolatory line on the calibration curve.

## Results

### Sample Morphology

**Figure 3:**
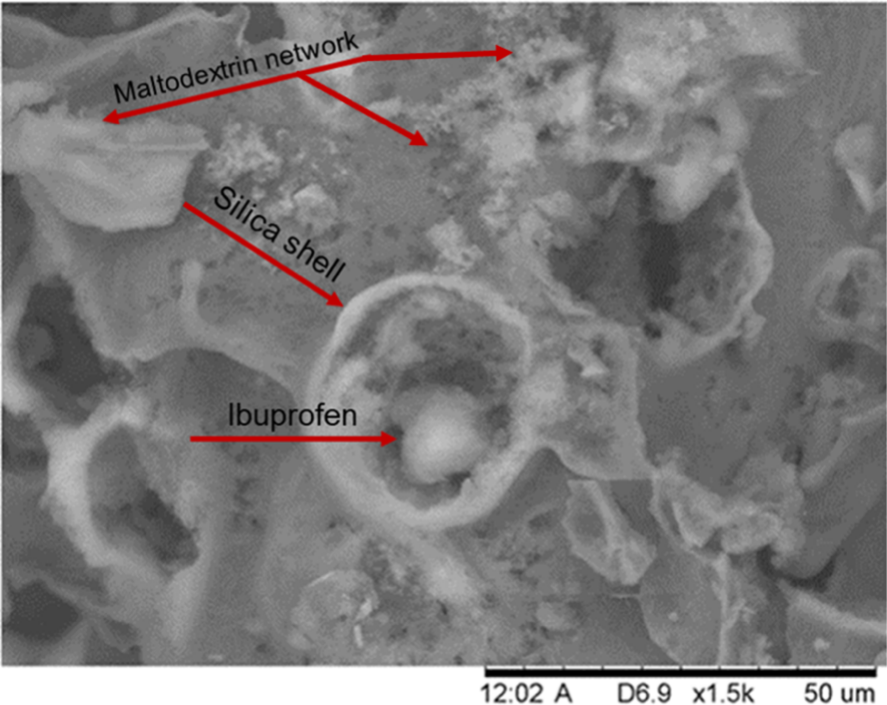
SEM image of freeze-dried maltodextrin containing an “antibubble” filled with a network of silica containing small drug particles. With the arrows, we identify the maltodextrin network where the silica shells are located and where the ibuprofen is encapsulated.

In Figure 3, the SEM image of the freeze-dried system obtained using cyclooctane is depicted. Here, we note that the freeze-dried maltodextrin contains polydispersed antibubbles, with an average size of 200 μm, filled with a network of R972.

### TGA and DTG Analysis

**Figure 4:**
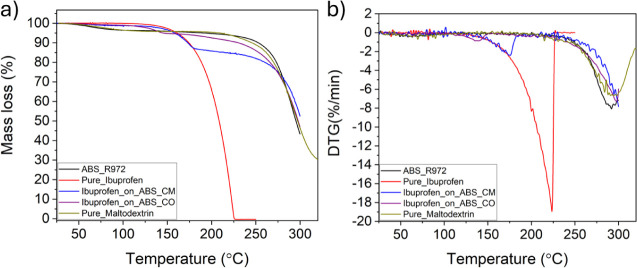
(a) Thermogravimetric analysis and (b) first derivatives (DTG) of ibuprofen encapsulated in the antibubbles (Ibuprofen_on_ABS_CM and Ibuprofen_on_ABS_CO), compared with empty antibubbles (ABS_R972), Pure_Maltodextrin, and Pure_Ibuprofen.

Thermogram of ABS_R972, Pure_Ibuprofen, Ibuprofen_on_ABS_CM, Ibuprofen_on_ABS_CO, and Pure_Maltodextrin and their first derivatives (DTG) are shown in Figure 4. The thermogravimetric curves obtained in triplicate were reproducible and showed distinctive weight loss on heating. However, some similarities were also observed, allowing for the identification of three regions of interest (ROI).

The first ROI, between 25 and 100 °C, is associated with water evaporation. In this region, Pure_Ibuprofen showed no mass loss, while Pure_Maltodextrin and ABS_R972 had mass losses of 3.2%, and Ibuprofen_on_ABS_CM and Ibuprofen_on_ABS_CO showed a similar mass loss of 1%.

In the second ROI, between 100 and 225 °C, ABS_R972 remained stable and, in agreement with the literature,^29−31^ the onset of the thermal degradation and the full decomposition of Pure_Ibuprofen were observed at 125 and 225 °C, while the thermal decomposition of maltodextrin started near 220 °C. Additionally, around 120 °C, a mass loss of 5.5% was detected for Ibuprofen_on_ABS_CO, while for Ibuprofen_on_ABS_CM, this value was 13% at 125 °C. This is an interesting result, showing that for both systems, drug loading corresponds to the theoretical mass of the drug. Besides, from the DTG curves in the second ROI (Figure 4B), the onset of drug degradation in Ibuprofen_on_ABS_CM is closer to that of Pure_Ibuprofen, while for Ibuprofen_on_ABS_CO, an earlier onset occurs. This is experimental evidence of our visual observation that when using CO, there is a change in the ibuprofen crystallinity.

Finally, in the third ROI, between 225 and 310 °C, a shift to higher temperatures of maltodextrin degradation was observed for Ibuprofen_on_ABS_CM and Ibuprofen_on_ABS_CO. This might indicate some interaction between the drug and the carbohydrate matrix. As expected, the degradation of ABS_R972 and Pure_Maltodextrin persists in this temperature range.

### Evolved Gas Analysis: QMS-FTIR

**Figure 5:**
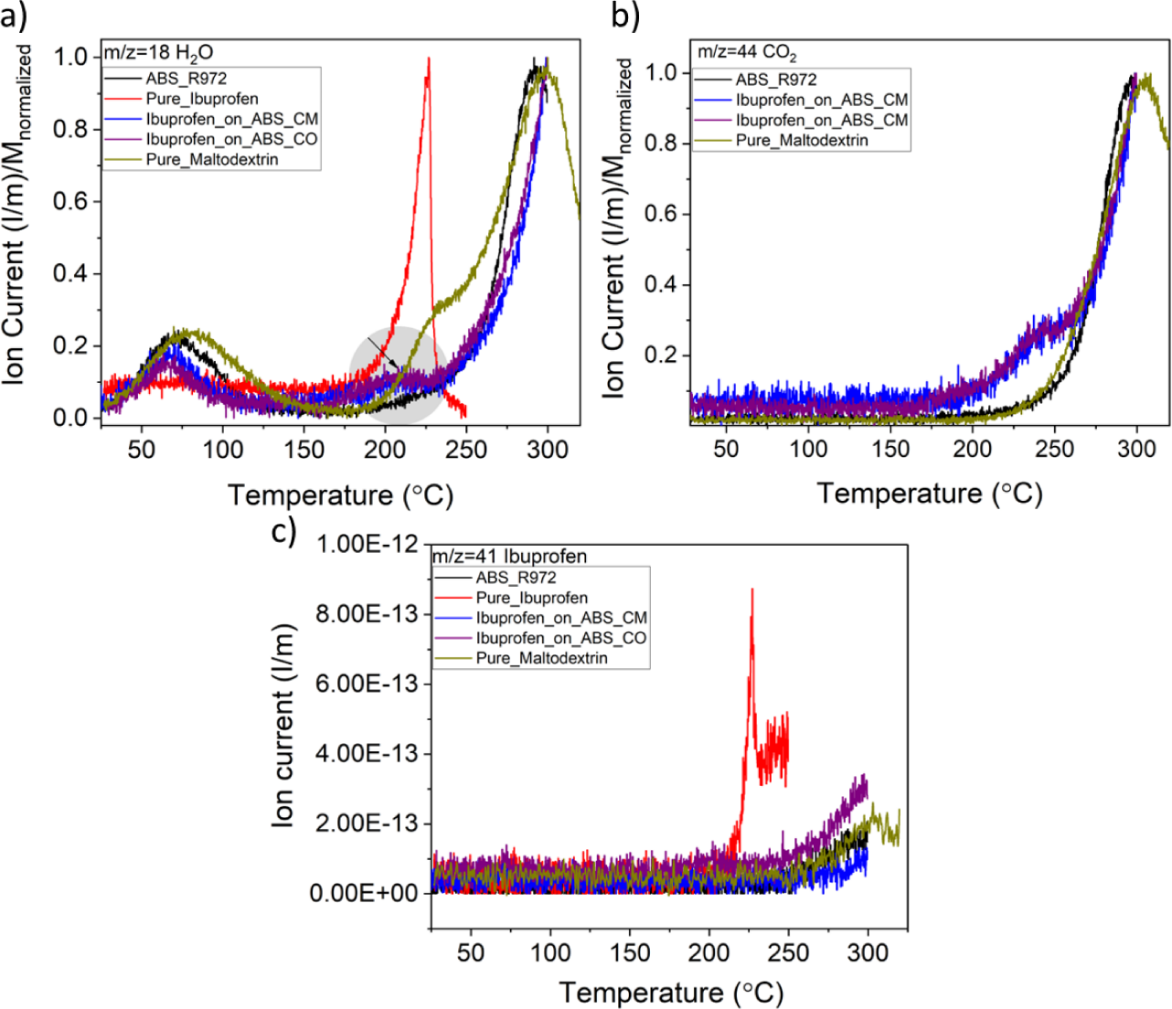
(a) Ion current for *m*/*z* = 18 showing the emission of water related to the evaporation of moisture under 100 °C and around the decomposition temperature of the pure drug, as highlighted in gray. (b) Mass spectrum for *m*/*z* = 44 showing that CO_2_ emission attributed to maltodextrin and ABS_R972 is also observed in the encapsulated systems at distinct ROIs. (c) Mass spectrum for *m*/*z* = 41, related to the loss of HCOOH in Pure_Ibuprofen, is observed at the second ROI. For better comparison, the mass spectrum for water and CO_2_ were normalized first on the sample mass and then to unity, while the signal at *m*/*z* = 41 was only normalized to mass.

Figure 5 shows the distribution of the ion current of the gaseous products from the samples’ thermal degradation, displaying signals with *m*/*z* = 18 (water, Figure 5A), *m*/*z* = 44 (CO_2_, mostly due to the degradation of the sugar, Figure 5B), and *m*/*z* = 41 (attributed to the C=O emission of the carboxyl (COOH) due to the degradation of the ibuprofen molecule and related to a strong emission at *m*/*z* = 161, Figure 5C).^36^

Looking at the first ROI of Figure 5A, the signal is dominated by surface water evaporation and moisture desorption. However, the center of the peaks differs across samples; for Pure_Maltodextrin, it is located at 80 °C, shifted to 72 °C for ABS_R972, and to even lower temperatures, i.e., 70 °C, for both Ibuprofen_on_ABS_CM and Ibuprofen_on_ABS_CO. This decrease in the water evaporation temperature can be attributed to differences in the binding interaction and location of the water molecules in the samples. In the second ROI, the water signal at 225 °C is related to the decomposition of Pure_Ibuprofen. This observation also allows for a further comparison of the shape of the *m*/*z* signals between ABS_R972, Ibuprofen_on_ABS_CM, and Ibuprofen_on_ABS_CO around 200 °C, where we also note an indication of drug degradation as highlighted in gray. Furthermore, as shown in Figure 5B, while emission from the CO_2_ moieties present in maltodextrin and ABS_R972 is detected in the third ROI, Ibuprofen_on_ABS_CM and Ibuprofen_on_ABS_CO also show loss of CO_2_ in the second ROI. Finally, although the signal from the carboxyl group assigned to the pure drug as seen in the second ROI in Figure 5C could not be identified in Ibuprofen_on_ABS_CM nor in Ibuprofen_on_ABS_CO due to the low quantity of encapsulated drug and the detection limit of the instrument, molecular vibrations assigned to ibuprofen were observed in the FTIR spectra of the evolved gas analysis (EGA), as discussed in the next section.

**Figure 6:**
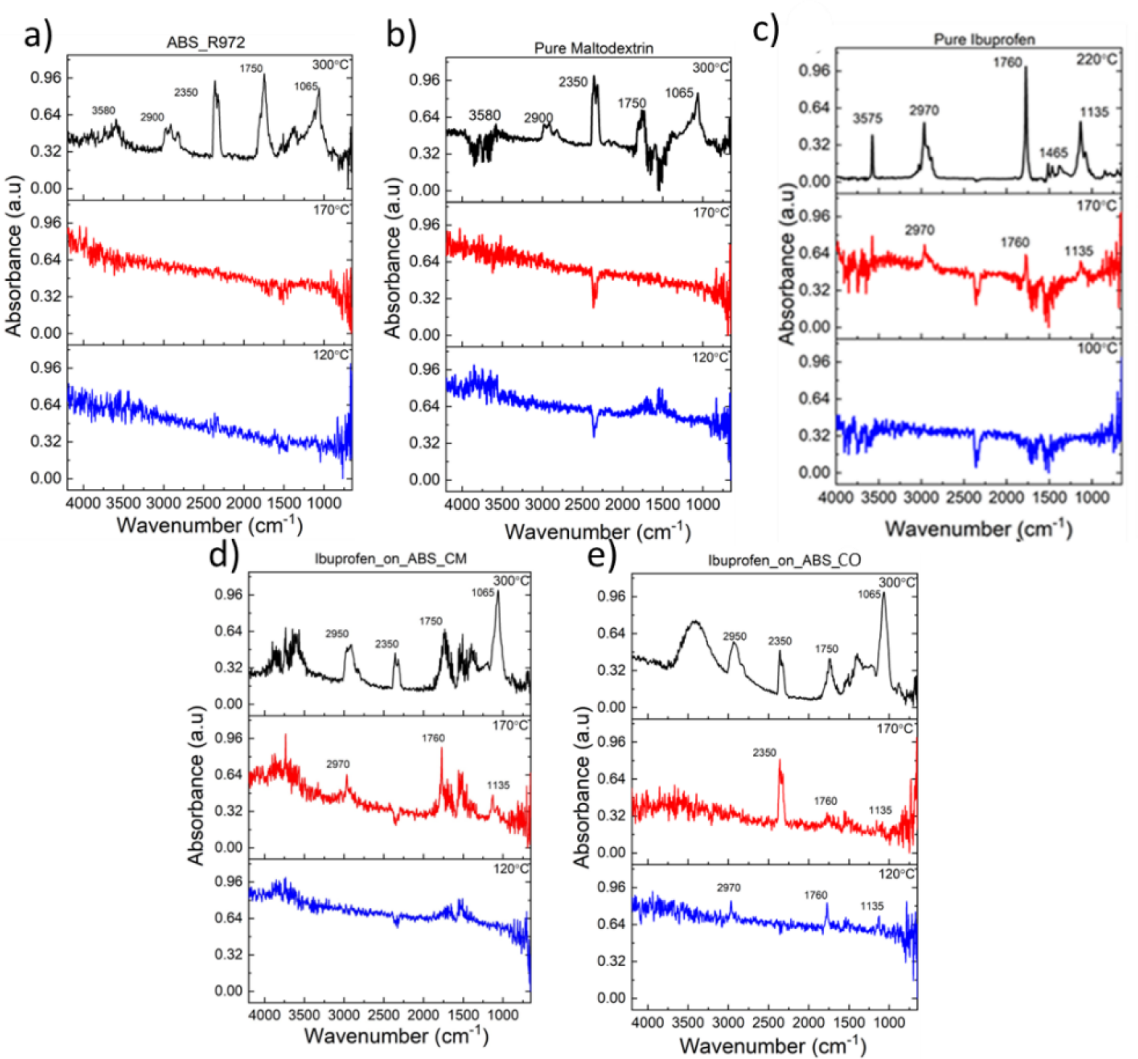
FTIR spectra for the 5 samples. (a) ABS_R972 shows mostly the maltodextrin degradation at 300 °C. (b) Pure_Maltodextrin shows carbohydrate degradation at 300 °C. (c) Pure_Ibuprofen shows a higher absorption intensity at 220 °C related to the faster rate of degradation observed in the DTG curve. (d) Ibuprofen_on_ABS_CM shows a small emission at 170 °C and (e) Ibuprofen_on_ABS_CO at 120 °C. Note that water and carbon dioxide signals were over subtracted from the beginning of the measurement in (b). This common artifact is related to the presence of these gases in the TGA furnace.

The measured FTIR gas spectra for ABS_R972 and Pure_Maltodextrin, as shown in Figure 6A,B, confirm that water (vibration at 3580 cm^–1^) and carbon dioxide (vibration at 2350 cm^–1^) are released on heating. However, the interesting part of the data that can be used to fully describe the degradation process is above 120 °C, where at the highest temperature, the spectra of ABS_R972, Figure 6A, basically mimic those of Pure_Maltodextrin, Figure 6B. For both samples, the broad absorbance band at 3580 cm^–1^ corresponds to the stretching vibrations of O–H bonds, typically present in the hydroxyl groups of glucose units; the band at 2900 cm^–1^ corresponds to the stretching vibrations of C–H bonds in the aliphatic moiety of the molecule; the band at 1750 cm^–1^ to the stretching vibrations of C=O bonds in the carbohydrate; and the band at 1065 cm^–1^ to various C–O stretching vibrations, including the glycosidic linkages between glucose units.^37−39^ For ibuprofen, Figure 6C, a vibration at 1760 cm^–1^, assigned to C=O stretching from the carbonyl group, and a hint of the complex band at 2970 cm^–1^, corresponding to the aliphatic C–H stretches^1^ are observed at 170 °C. From the spectra at 220 °C, selected due to its higher absorbance, we notice a sharp band at 3575 cm^–1^, attributed to the O–H stretching in the carboxylic acid group, a very clear band at 2970 cm^–1^, a sharp band at 1760 cm^–1^ assigned to the stretching vibration of the carbonyl group (C=O) in the carboxylic acid moiety of Ibuprofen, and a broad peak at 1135 cm^–1^ that might correspond to C–O–H stretching vibrations in different parts of the molecule. Additionally, three small peaks at 1465 cm^–1^, 1384 cm^–1^, and 1515 cm^–1^, often associated with aromatic C–C–H bending vibrations, a combination of C–H bending and C–C stretching vibrations in the aromatic ring, and C=C stretching vibrations in the aromatic ring, respectively, are also detected.^40−42^ Finally, by connecting the TG, MS, and FTIR results, Figures 4, 5, and 6, it is possible to determine that in the second ROI, between 100 and 225 °C, the three bands at 2970, 1760, and 1135 cm^–1^ assigned to Pure_Ibuprofen (Figure 6C) emerge at 170 °C in Ibuprofen_on_ABS_CM (Figure 6D) and at 120 °C in Ibuprofen_on_ABS_CO (Figure 6E). As expected, due to the encapsulation and the amount of sample, the vibrations are less intense when compared to those represented in Figure 6C, while the shift of ibuprofen degradation when encapsulated in Ibuprofen_on_ABS_CO corroborates again with the idea that the encapsulation process modified the drug morphology. Additionally, an alteration to the C–H bonds is clearly reflected in the FTIR data. For instance, the vibration observed at 2970 cm^–1^ for Pure_Ibuprofen, Figure 6C, and at 2900 cm^–1^ on ABS_R972, Figure 6A, shifts to 2950 cm^–1^ in the encapsulated systems. Table 1 summarizes the mass loss percentage and associates the molecular group dissociation with thermal decomposition in each ROI.

**Table 1:**
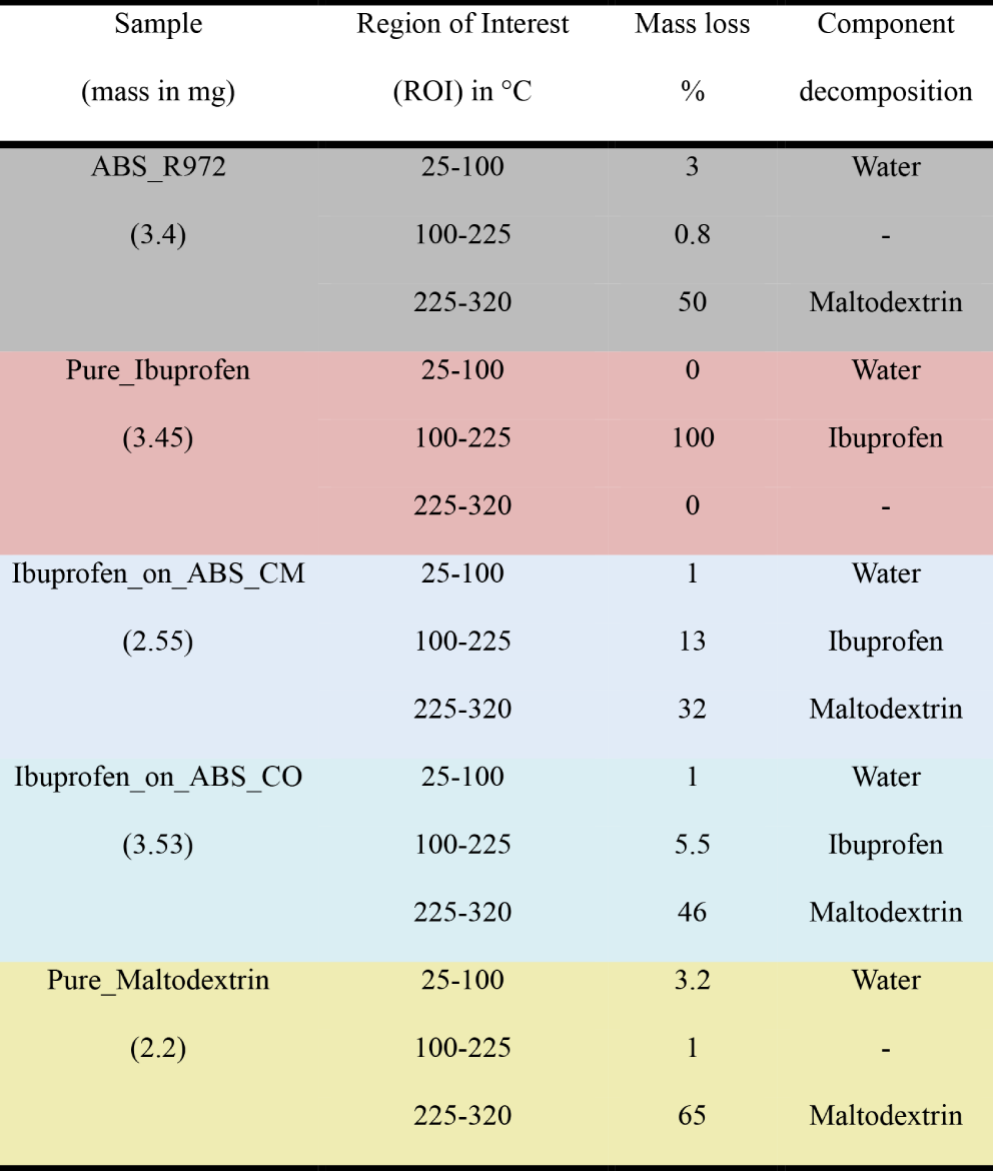
Mass Loss and Assignment of the Molecular Decomposition Obtained from the Evolved Gas Analysis for ABS_R972, Pure_Ibuprofen, Ibuprofen_on_ABS_CM, Ibuprofen_on_ABS_CO, and Pure_Maltodextrin^a^

a Based on the composition of the samples, Ibuprofen_on_ABS_CM contains about 10.3% and Ibuprofen_on_ABS_CO about 5.4% Ibuprofen.

### Differential Scanning Calorimetry (DSC) and X-Ray Powder Diffraction (XRPD)

**Figure 7:**
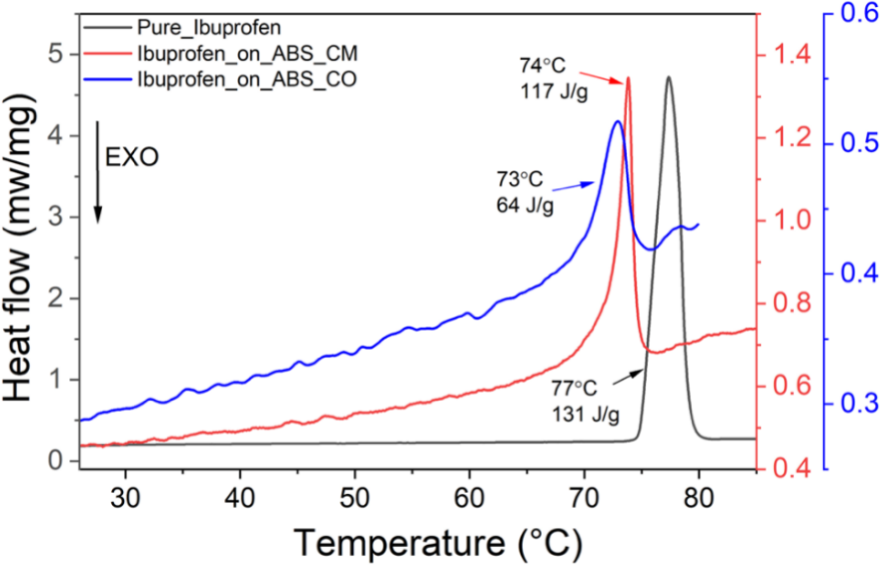
Differential scanning calorimetry measurements for Pure_Ibuprofen (black line), Ibuprofen_on_ABS_CM (red line), and Ibuprofen_on_ABS_CO (blue line). The results show a strong endothermic peak at 77 °C for Pure_Ibuprofen, at 74 °C for Ibuprofen_on_ABS_CM and at 73 °C for Ibuprofen_on_ABS_CO. The full range of the thermograms are presented in Figure S5. Note that for clarity each sample has a unique *y*-axis.

DSC curves for Pure_Ibuprofen, Ibuprofen_on_ABS_CM, and Ibuprofen_on_ABS_CO are shown in Figure 7. For Pure_Ibuprofen (black lines), the melting point, determined by the maximum of the endothermic peak, is observed at 77 °C, with the enthalpy of the transition, Δ*H*, equal to 131 J/g. This agrees with previous reports on the thermodynamic aspects of racemic Ibuprofen.^43^ On the other hand, for Ibuprofen_on_ABS_CM, the melting point is found at 74 °C, and for Ibuprofen_on_ABS_CO, it is shifted to 73 °C.

Using the ibuprofen mass obtained from the TGA analysis and reported as a percentage of the total sample mass in Table 1, the enthalpy of the transition was calculated for Ibuprofen_on_ABS_CM, using an area from 60 to 83 °C, and for Ibuprofen_on_ABS_CO, an area from 60 to 76 °C. The obtained Δ*H* equals 117 and 64 J/g, respectively. The shift of the melting point to lower temperatures is a characteristic observation for encapsulated drugs,^44−47^ while the lower enthalpy is yet another indication of a modification of the crystallinity, with Ibuprofen_on_ABS_CO being the least crystalline.^48,49^

In this context, X-ray diffraction analysis is very important since changes in the crystal structure can be readily verified by comparing the experimental diffractograms to the calculated ones using CIF files, Figures S6 and S7.^50^

**Figure 8:**
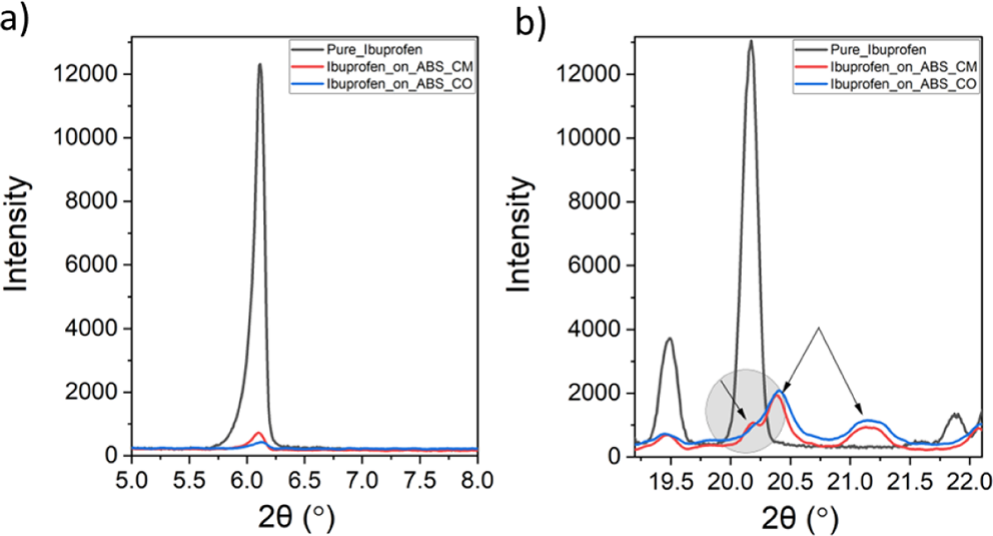
XRPD patterns for Pure_Ibuprofen, Ibuprofen_on_ABS_CM, and Ibuprofen_on_ABS_CO. (a) Between 5° and 8°, the decrease of the Bragg reflection intensity indicates a successful encapsulation. (b) The observation of new reflections with different peak shapes in the range of 19°–22° confirms that new crystalline forms were obtained (as arrows shown at 20.2°, 20.4°, and 21.2°); see also Figure S7.

In Figures 8A,B, we compare the XRPD pattern of Pure_Ibuprofen to Ibuprofen_on_ABS_CM and Ibuprofen_on_ABS_CO in two ROIs. In the case of Pure_Ibuprofen, Figure 8A, the position of the Bragg reflections is in agreement with the reported racemic form,^51−55^ while the decrease in intensity of the Bragg reflection at 6.1° (2θ) observed for Ibuprofen_on_ABS_CM and Ibuprofen_on_ABS_CO is a clear indication that ibuprofen was successfully encapsulated.^56^ Besides, the observation of new reflections at 20.4° and 21.3° (2θ), indicated by arrows in Figure 8B, confirms conversion to a new form. Furthermore, the broadening of these reflections indicates a certain loss of crystallinity. Moreover, the differences in the shape of these Bragg reflections, where a clear shoulder is observed for Ibuprofen_on_ABS_CM at 20.2° (2θ), also confirm differences in crystallinity between the encapsulated drugs. The amorphous nature of maltodextrin, ABS_R972, and silica R972 is evidenced by their broader X-ray patterns shown in Figure S6. This result is also in agreement with the relevant literature.^51,57^

Using eq 1 and the data reported in Figure S6, the relative crystallinity, *R*_C_, given by the ratio of the crystalline area (*A*_C_, defined as the area under the Bragg reflections) to the total area under the diffractogram (*A*_T_)^58^ was obtained for the encapsulated systems. In full agreement with the Δ*H* values, these results show that the amorphous contribution in Ibuprofen_on_ABS_CM is 16%, while for Ibuprofen_on_ABS_CO, this value is higher and equal to 25%.
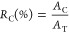
1

### Microscope Images and Raman Spectra

**Figure 9:**
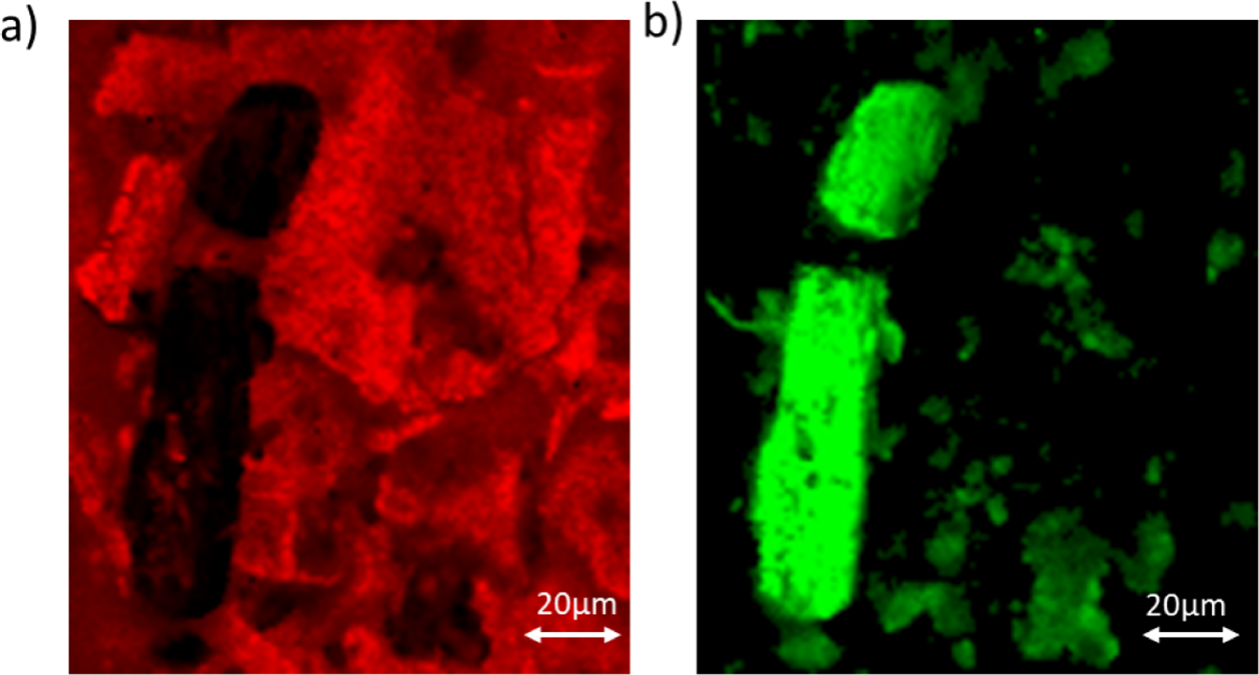
Raman maps showing the freeze-dried maltodextrin in red (a) covering an ibuprofen crystallite encapsulated in the antibubbles prepared using cyclomethicone D4. The crystallite is shown in green in (b).

**Figure 10:**
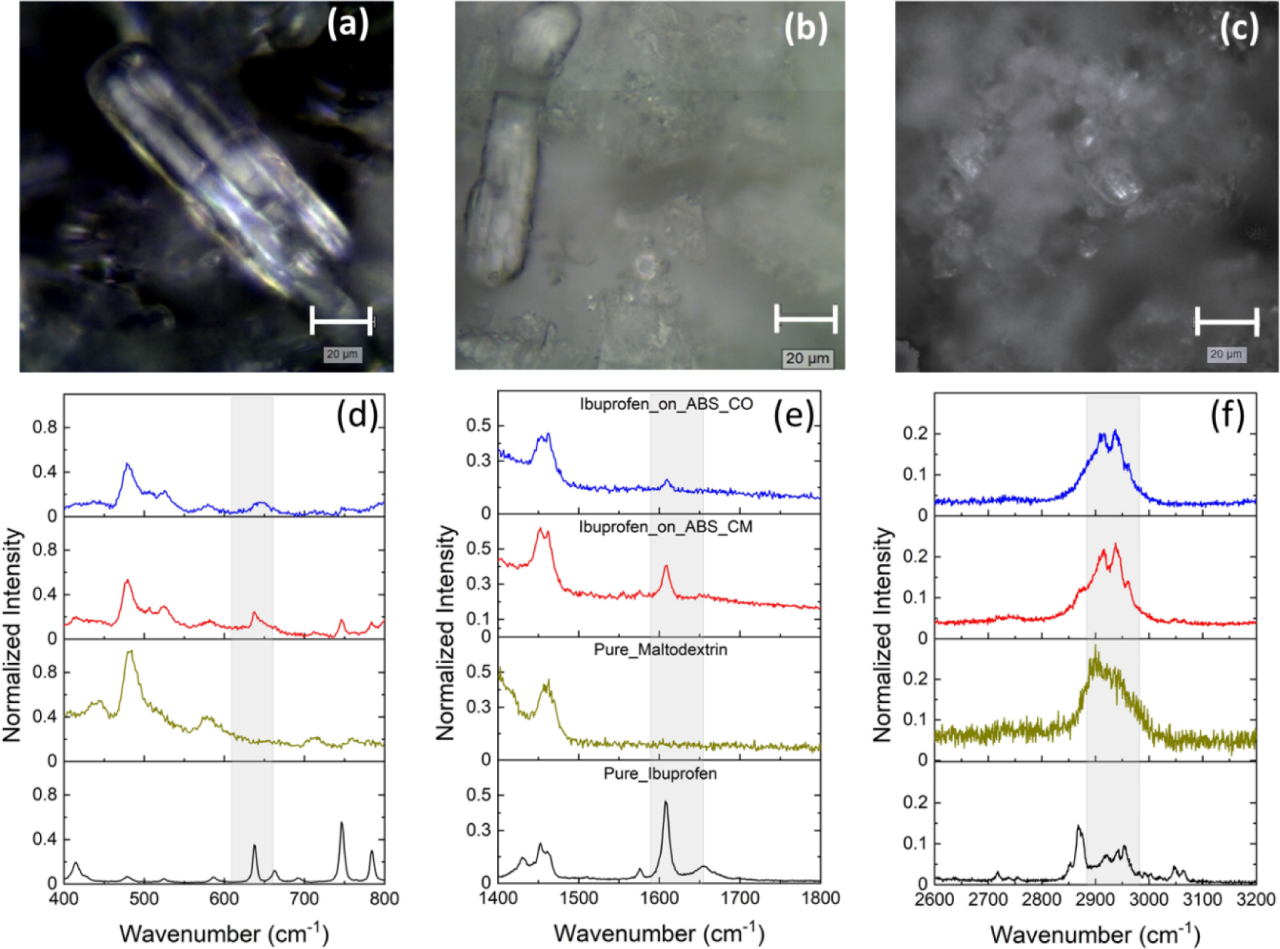
Visual light microscopy image representation of the free drug molecule (a) as well as the drug encapsulated in the R972 antibubbles: Ibuprofen_on_ABS_CM (b) and Ibuprofen_on_ABS_CO (c). Raman spectra for all samples are shown in d–f. All spectra were normalized to the highest internal vibration of each sample.

The Raman map for Ibuprofen_on_ABS_CM obtained by using PCA analysis, shown in Figure 9, gives spatial insight into the distribution of ibuprofen within the maltodextrin matrix. Furthermore, the detailed analysis of the microscope images from Pure_Ibuprofen, Ibuprofen_on_ABS_CM, and Ibuprofen_on_ABS_CO is shown in Figure 10A,B,C, respectively. Based on the scale bar, we identify that the length of the ibuprofen crystallites is about 65 μm.

The Raman spectra of maltodextrin (red color), Pure_Ibuprofen (black color), and the encapsulated drugs (Ibuprofen_on_ABS_CM and Ibuprofen_on_ABS_CO with blue and yellow colors, respectively) are shown in Figure 10. The spectra were normalized to the highest internal vibration of each sample and labeled with different colors to allow comparison of different regions, and thus a better analysis of the interaction between the drug molecule, the silica particles, and the sugar matrix. To distinguish changes at the intermolecular level between the encapsulated drugs and the relative pure substances of maltodextrin and ibuprofen, we divided the Raman spectrum into 3 different ROIs: 400–800 cm^–1^, Figure 10D, 1400–1800 cm^–1^, Figure 10E, and 2600–3200 cm^–1^, Figure 10F. For maltodextrin, one observes the C–C backbone symmetric stretching at 480 cm^–1^, C–O bending vibrations or ring deformations at 578 cm^–1^ and CH_2_ at 1457 cm^–1^. In the second ROI, as shown in Figure S5, we observed C–O and C–C stretching vibrations or H–C–H deformations.^59−61^ For Pure_Ibuprofen, the peak at 635 cm^–1^ is assigned to in-plane C=C ring deformation, the peak at 746 cm^–1^ to C=O and aromatic C–H stretching, and the strong vibrations at 1607 cm^–1^ to C–H vibration of the aromatic ring and to the C=C carboxyl group.^62^ In the third ROI, we observed three peaks in the Pure_Ibuprofen spectrum at 2914, 2937, and 2954 cm^–1^, which reflect the C–H bonds. These bands have very small intensities in maltodextrin.

The Raman spectra within the 400–1800 cm^–1^ region are the fingerprint region of a molecule and often denote differences between ibuprofen in the glassy and pure crystalline states.^62,63^ Thus, from the analysis of the marked spectral regions of Figure 10D,E (gray color), it is clear that the strong signals at 635 and 1607 cm^–1^ in Pure_Ibuprofen are weaker and broader in the encapsulated drugs. We also note that the mode at 1607 cm^–1^ shifted to 1610 cm^–1^ in the Ibuprofen_on_ABS_CM and Ibuprofen_on_ABS_CO spectra. This shift is, however, very small when compared to observations for other pharmaceutical compounds after encapsulation within a silica framework.^64^ Moreover, modification of the C=O stretching at 1655 cm^–1^ further confirms that the spectral changes are related to the amorphization of the drug molecule. A similar signal was also observed when a core–shell structure of ibuprofen crystals was formed via nonclassical crystallization.^65^ Such an approach is also strategically utilized to enhance drug dissolution. To conclude, these alterations solely indicate a perturbation of the internal structure of the drug encapsulated inside the antibubbles, as already evidenced by the thermal analysis and XRPD results, without hydrogen bonding with the silanol group of the silica matrix as observed when using the sol–gel method.^64^ Additionally, by comparing the Raman spectra of Ibuprofen_on_ABS_CO and Ibuprofen_on_ABS_CM, we can observe a clear difference between their glassy-like states, as the fingerprint region of the more amorphous Ibuprofen_on_ABS_CO shows lower intensities and broader bands.

### Zeta Potential

Pure silica R972 showed a zeta potential of −30.82 mV, while for ABS_R972, the value equals −17.52 mV. This confirms lower electrostatic repulsion between the antibubbles, and can help maintain stability and prevent aggregation.^66−69^ After encapsulating Pure_Ibuprofen, which in this work has a zeta potential equal to −10.56 mV, in silica antibubbles, the surface charge for both samples became closer to the value of the ABS_R972, with Ibuprofen_on_ABS_CM showing a zeta potential of −18.57 mV and Ibuprofen_on_ABS_CO showing a value of −16.34 mV. These values are close to the ones reported for ibuprofen loaded in menthosomes.^70^

### In Vitro Dissolution Studies

Dissolution profiles of the Pure_Ibuprofen, Ibuprofen_on_ABS_CM, and Ibuprofen_on_ABS_CO show small differences between Pure_Ibuprofen and Ibuprofen_on_ABS_CM, while increasing the amorphous part of ibuprofen in Ibuprofen_on_ABS_CO caused a slight increase in its dissolution.^71^

## Discussion and Conclusions

The encapsulation of ibuprofen within Pickering-stabilized antibubbles prepared using either an S/O/W or an O/W emulsion, provides an easy method that offers rapid dissolution, drug loads that match the theoretically calculated value, and might have the potential to be used both in targeted delivery and taste masking.

Successful encapsulation was demonstrated by the visual representation of the drug’s distribution using Raman confocal microscopy, specifically the 2D mapping technique, which allowed for showing the ibuprofen crystallites absorbed in the sugar matrix. Moreover, the intricate interplay among the drug, the maltodextrin matrix, and the silica particles was explained through a multifaceted analytical approach that combined thermogravimetric (TG) analysis coupled with mass spectrometry (MS) and Fourier transform infrared spectroscopy (FTIR). Distinct weight loss patterns in various temperature intervals indicated differences in physically adsorbed water, volatile functional groups, and recalcitrant structures. In more detail, DSC analysis showed a shift in the melting point of our systems, 77 °C for Pure_Ibuprofen compared to 74 °C for Ibuprofen_on_ABS_CM and 73 °C for Ibuprofen_on_ABS_CO, while the measured enthalpy dropped for the latter. Dissimilar decomposition rates and peak patterns, observed in the thermal decomposition step between 100 and 225 °C, imply changes in the thermodynamic interaction between ibuprofen and the encapsulating matrix. This interplay adds a layer of complexity to the understanding of the encapsulation process, which was explained by evolved gas analysis (EGA). This chemical perspective reveals the gaseous products released during thermal degradation, and the identification of H_2_O, CO_2_, and ion emission bands coupled with specific absorption bands corresponding to ibuprofen degradation offered a chemical fingerprint of the transformations occurring at different temperature intervals.

From the FTIR analysis, by comparing the gas spectra of Pure_Ibuprofen at 220 °C to those of Ibuprofen_on_ABS_CM at 170 °C and Ibuprofen_on_ABS_CO at 120 °C, characteristic vibrations of the pure drug are observed. Therefore, we argue that drug degradation starts at slightly different temperatures. While based on the differential scanning calorimetry (DSC) and X-ray powder diffraction (XRPD) patterns, it is shown that the use of the oils caused the amorphization of the drug in both antibubbles. Additionally, comparison of the Raman spectra of the two encapsulated drugs with Pure_Ibuprofen and Pure_Maltodextrin showed a shift in some vibrational modes and a double peak at 880 cm^–1^, indicating an intermolecular change of the ibuprofen structure in the encapsulated systems. However, there is no spectral indication of a potential interaction between the drug molecule and the antibubble.

To obtain information concerning the stability of the prepared samples, their zeta potential was determined by the electrophoretic mobility technique. Overall, the zeta potential indicates that ibuprofen encapsulation in Pickering antibubbles has a stabilizing effect on the ibuprofen particles, which might promote better colloidal stability compared to the pure drug. Finally, ibuprofen-loaded Pickering antibubbles exhibited similar or slightly enhanced dissolution rates in comparison to those of crystalline ibuprofen. As a next step, a careful pharmacokinetic experiment should be envisaged, and the data should be compared with bioavailability results obtained for, among others, ibuprofen encapsulated in biocompatible metal–organic frameworks,^5^ sporopollenin exine capsules (SEC),^72^ and dye-drug mixed micelle formulations.^73^

In conclusion, the combination of the selected advanced analytical techniques provided a complete understanding of ibuprofen encapsulation within antibubbles. Our findings also highlight the significance of the proposed approach for a comprehensive evaluation of the molecular properties of encapsulated pharmaceutical forms.

## Supporting Information Available

The Raman data for Pure_Ibuprofen, Pure_Maltodextrin, Ibuprofen_on_ABS_CM, and Ibuprofen_on_ABS_CO collected with gratings of 600 lines/mm and 1200 lines/mm (Figures S1–S4); the DSC data between −100 and 100 °C for Pure_Ibuprofen, Ibuprofen_on_ABS_CM, and Ibuprofen_on_ABS_CO (Figure S5); comparison of the XRPD patterns obtained for Pure_Ibuprofen, Ibuprofen_on_ABS_CM, and Ibuprofen_on_ABS_CO with the calculated pattern of the racemic ibuprofen (Figures S6 and S7) (PDF)
