# Supplementary material for: Encapsulation of Ibuprofen by Pickering-Stabilized Antibubbles
Source: ACS Omega. 2025 Jan 22;10(7):7131–41. doi: 10.1021/acsomega.4c10244 (PMC11866197; doi:10.1021/acsomega.4c10244)
Supplement: Supplementary file 1 — ao4c10244_si_001.pdf [file ao4c10244_si_001.pdf]

Encapsulation of Ibuprofen by Pickering-Stabilized Antibubbles

Charalampos Tsekeridis[1*], *Paloma Manuelle Marques da Silva*[1], *Guilherme B. Strapasson*[2]

Albert T. Poortinga[3] *and Heloisa Nunes Bordallo*[1]

[1]Niels Bohr Institute, University of Copenhagen, DK-2100, Copenhagen, Denmark

[2]Department of Chemistry and Nanoscience Center, University of Copenhagen, Denmark

[3]Department of Mechanical Engineering, Polymer Technology, Eindhoven University of Technology, Eindhoven, the Netherlands

Raman Data for the Pure_Ibuprofen, Pure_Maltodextrin and Ibuprofen_on_ABS_CM using the 785nm laser excitation and 2 different gratings, at 600 lines/mm and 1200 lines/mm.

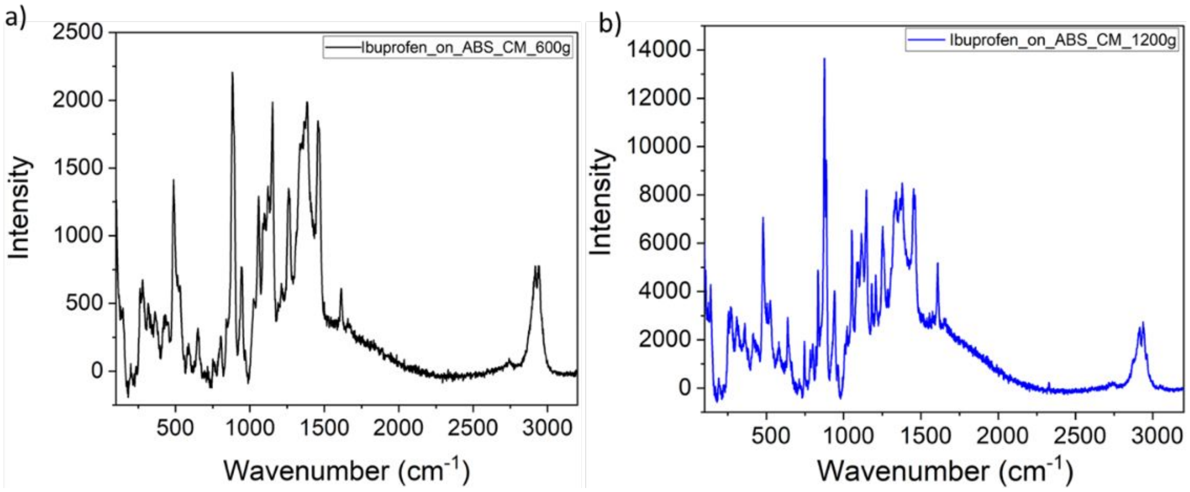

Figure S1. Raman spectra using a grating of 600 lines/mm (a) and 1200 lines/mm (b) for Ibuprofen_on_ABS_CM.

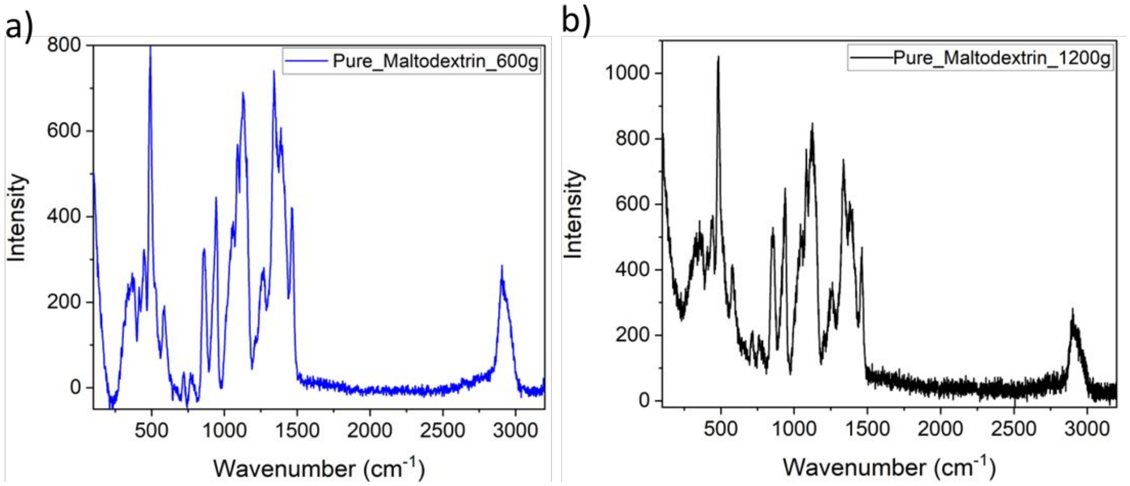

Figure S2. Raman spectra using a grating of 600 lines/mm (a) and 1200 lines/mm (b) for Pure_Maltodextrin.

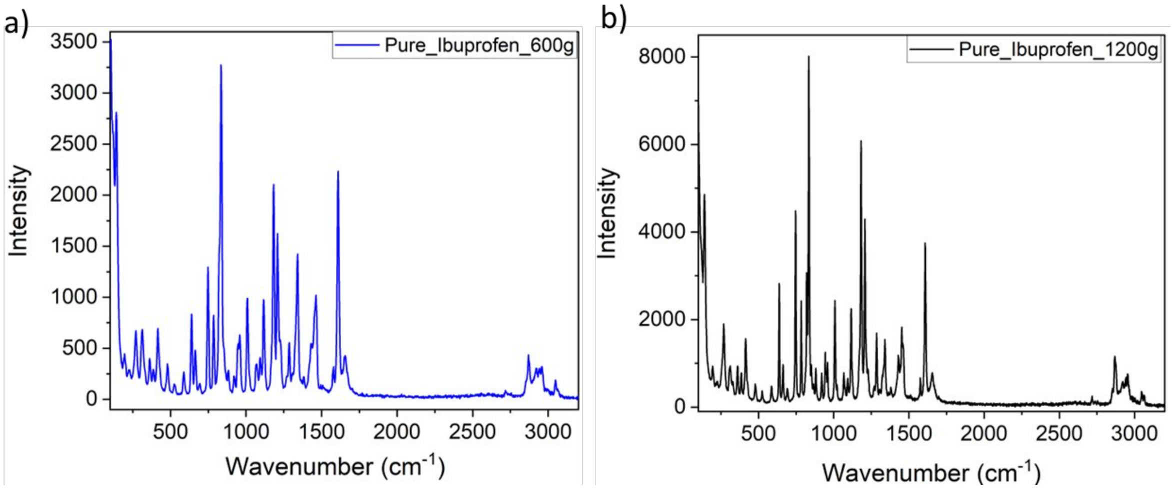

Figure S3. Raman spectra using a grating of 600 lines/mm (a) and 1200 lines/mm (b) for Pure_Ibuprofen.

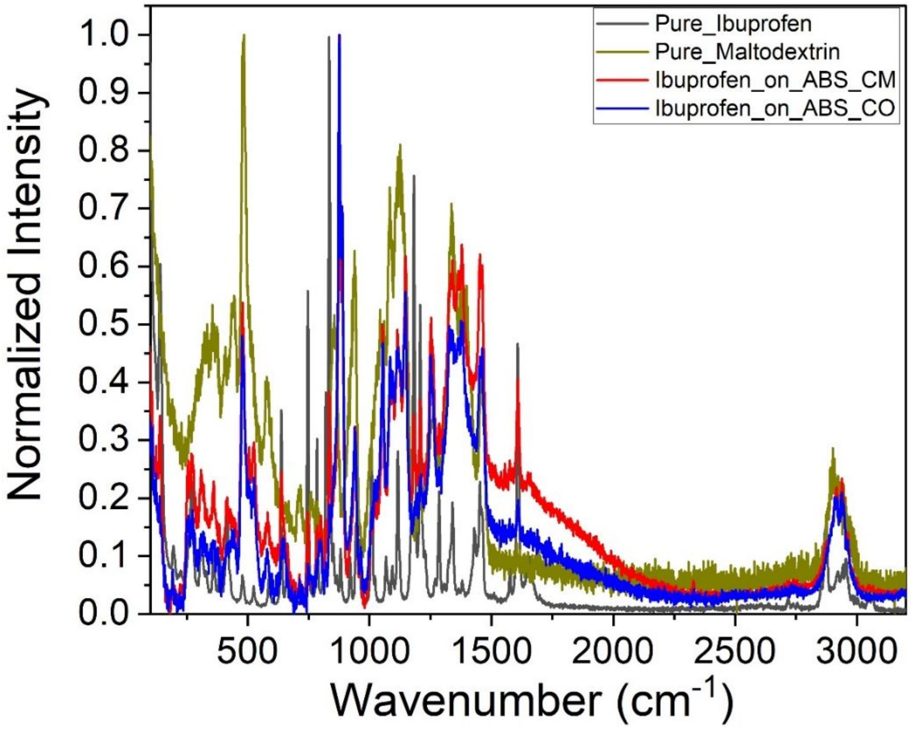

Figure S4. Full range of the Raman spectra for Pure_Ibuprofen, Pure_Maltodextrin, Ibuprofen_on_ABS_CM and Ibuprofen_on_ABS_CO.

DSC Data: Full range of measurements for Pure_Ibuprofen, Ibuprofen_on_ABS_CM and Ibuprofen_on_ABS_CO between -100 and 100 ˚C.

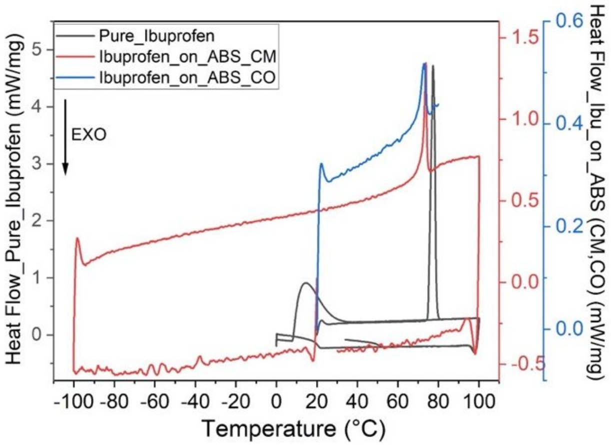

Figure S5 Full range of the DSC measurement for the three samples.

XRPD Data: Figure S6 shows the diffraction patterns for) Pure silica R972, ABS_R972 and Maltodextrin (a) Pure_Ibuprofen (b), Ibuprofen_on_ABS_CM (c) and Ibuprofen_on_ABS_CO (d). Figure S7 depicts experimental data together with the calculated pattern using the free version of the program Mercury[1] for the racemic Ibuprofen using data from reference.

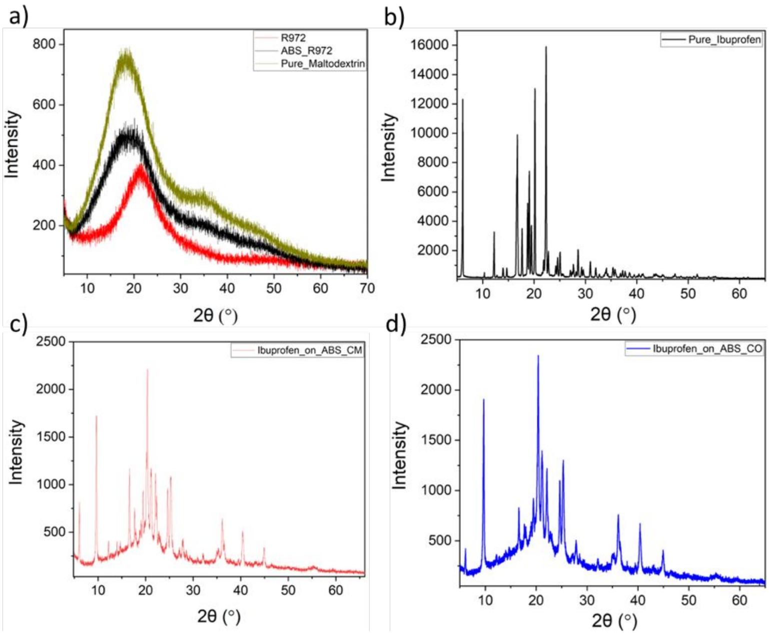

Figure S6. XRPD patterns for a) Pure silica R972, ABS_R972 and Maltodextrin b) Pure_Ibuprofen c) Ibuprofen_on_ABS_CM and d) Ibuprofen_on_ABS_CO.

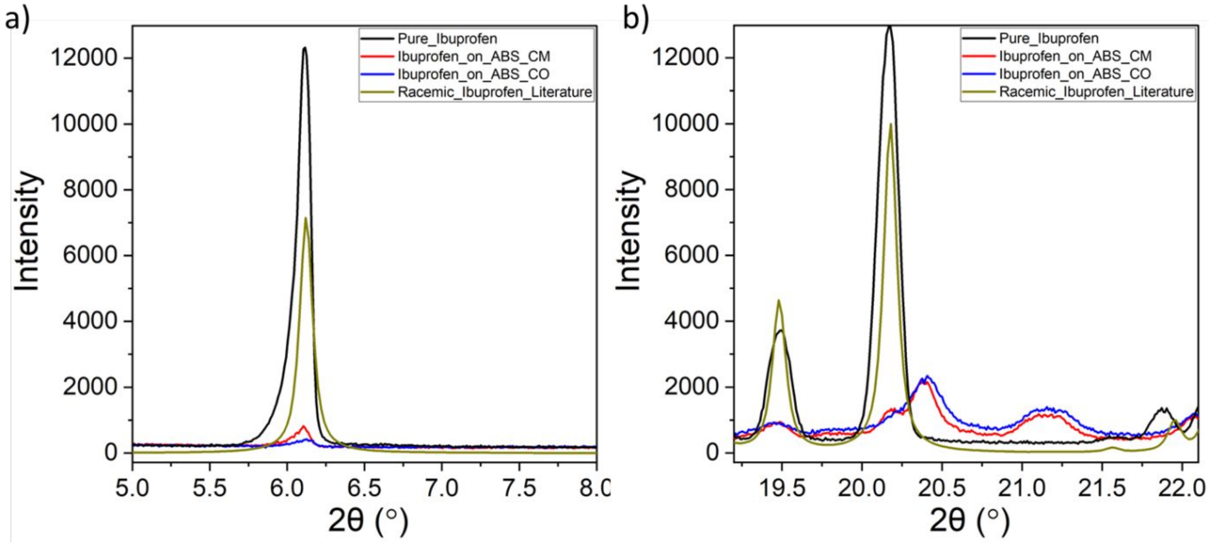

Figure S7. Experimental XRPD patterns for Pure_Ibuprofen, Ibuprofen_on_ABS_CM Ibuprofen_on_ABS_CO compared to the calculated pattern of the racemic Ibuprofen[2]. Diffraction pattern focusing on the reflections at $2\theta = 6.1^\circ$ (a) and $2\theta = 20.2^\circ$ (b).

References:

(1) Macrae, C. F.; Edgington, P. R.; McCabe, P.; Pidcock, E.; Shields, G. P.; Taylor, R.; Towler, M.; Van De Streek, J. *Mercury*: Visualization and Analysis of Crystal Structures. *J. Appl. Crystallogr.* **2006**, *39* (3), 453–457. https://doi.org/10.1107/S002188980600731X.
(2) Derollez, P.; Dudognon, E.; Affouard, F.; Danède, F.; Correia, N. T.; Descamps, M. *Ab Initio* Structure Determination of Phase II of Racemic Ibuprofen by X-Ray Powder Diffraction. *Acta Crystallogr. B* **2010**, *66* (1), 76–80. https://doi.org/10.1107/S0108768109047363.
